# Supplementary material for: Diverse and Synergistic Actions of Phytochemicals in a Plant-Based Multivitamin/Mineral Supplement against Oxidative Stress and Inflammation in Healthy Individuals: A Systems Biology Approach Based on a Randomized Clinical Trial
Source: Antioxidants (Basel). 2023 Dec 23;13(1):36. doi: 10.3390/antiox13010036 (PMC10812391; doi:10.3390/antiox13010036)
Supplement: Supplementary file 1 [file antioxidants-13-00036-s001.zip › Supplementary Tables.pdf]

Table S1. Comprehensive profiling of chemicals in PBS as identified by UPLC-Q-TOF-MS analysis.

No.	Compound name	Peak area	Chemical structure	MF[1]	MW	PubChem CID[2]	Excluded reason[3]
1	Proanthocyanidin derivatives	4.5	-	$C_{30}H_{25}O_{12}$	577.1	-	NS
2	Pantothenic acid	3967.2	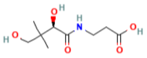	$C_9H_{17}NO_5$	219.2	6613	NP (Vitamins)
3	Cyanidin 3-O-rutinoside	633.2	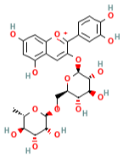	$C_{27}H_{31}O_{15}^+$	595.5	441674	-
4	Chlorogenic acid	4.5	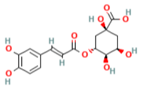	$C_{16}H_{18}O_9$	354.3	1794427	-
5	Gallocatechin-catechin-catechin	5.3	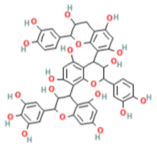	$C_{45}H_{38}O_{19}$	882.8	131752347	-
6	Procyanidin dimer	15.5	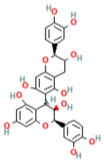	$C_{30}H_{26}O_{12}$	578.5	131752343	-
7	Tuberonic acid glucoside	6753.3	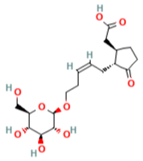	$C_{18}H_{28}O_9$	388.4	5281204	-
8	Catechin-gallocatechin-catechin	17.8	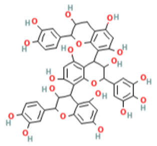	$C_{45}H_{38}O_{19}$	882.8	131752348	-
9	Epiafzelechin–epicatechin	4.5	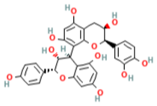	$C_{30}H_{26}O_{11}$	562.5	10239837	-
10	Procyanidin derivatives	9.3	-	$C_{30}H_{25}O_{12}$	577.1	-	NS
11	Quercetagetin-7-O-glucoside	641.8	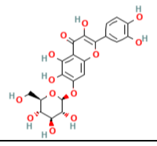	$C_{21}H_{20}O_{13}$	480.4	5320826	-

Table S1. (continued).

No.	Compound name	Peak area	Structure	MF[1]	MW	PubChem CID[2]	Excluded reason[3]
12	Procyanidin trimer	8.8	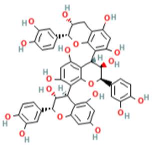	$C_{45}H_{38}O_{18}$	866.8	169853	-
13	Rutin	1151.2	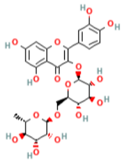	$C_{27}H_{30}O_{16}$	610.5	5280805	-
14	Kaempferol-3-O-rutinoside	6688.2	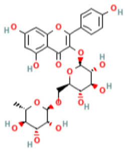	$C_{27}H_{30}O_{15}$	594.5	5318767	-
15	Q-3-Hex-Pen	15133.3	-	$C_{27}H_{31}O_{15}$	595.5	-	NS
16	Ellagic acid	4.5	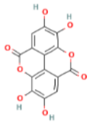	$C_{14}H_{6}O_{8}$	302.2	5281855	-
17	Quercetin 3-O-glucoside	1320.5	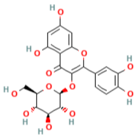	$C_{21}H_{20}O_{12}$	464.4	5280804	-
18	Sudachiin A	2450.2	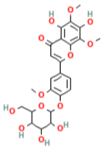	$C_{24}H_{26}O_{13}$	522.5	73829938	-
19	Quercetin-diglucoside	525.0	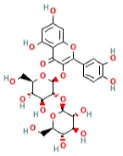	$C_{27}H_{30}O_{17}$	626.5	5282166	-
20	Isorhamnetin 3-O-glucoside	6603.2	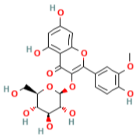	$C_{22}H_{22}O_{12}$	478.4	5318645	-
21	Quercitrin	4.5	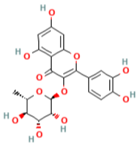	$C_{21}H_{20}O_{11}$	448.4	5280459	-
22	Naringin	6452.7	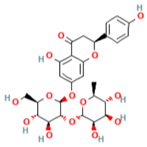	$C_{27}H_{32}O_{14}$	580.5	442428	-

Table S1. (continued).

No.	Compound name	Peak area	Structure	MF	MW	PubChem CID	Excluded reason
23	Hesperidin	7257.3	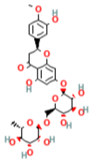	$C_{28}H_{34}O_{15}$	610.6	10621	-
24	Dicaffeoylquinic acid	9.5	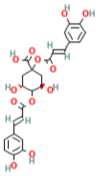	$C_{25}H_{24}O_{12}$	516.4	12358846	-
25	Rosmarinic acid	14619.2	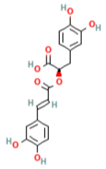	$C_{18}H_{16}O_{8}$	360.3	5281792	-
26	Petunidin glycoside glucuronide	3431.3	-	$C_{32}H_{31}O_{15}$	655.6	-	NS
27	Glucosyl–rhamnosyl isorhamnetin	4.5	-	$C_{31}H_{27}O_{14}$	623.1	-	NS
28	Pelargonidin-disacharide (hexose+pentose) acylated with acetic acid	298.2	-	$C_{28}H_{31}O_{15}$	607.5	-	NS
29	Kaempferol-hexose-pentose	62.7	-	$C_{28}H_{35}O_{14}$	595.6	-	NS
30	Quercetin	47293.8	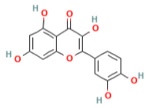	$C_{15}H_{10}O_{7}$	302.2	5280343	-
31	Peonidin 3-glucoside	530.3	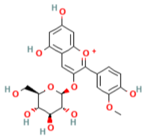	$C_{22}H_{23}O_{11}^{+}$	463.4	443654	-
32	Phloretin	4.5	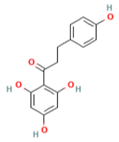	$C_{15}H_{14}O_{5}$	274.3	4788	-
33	Demethoxycurcumin	1068.0	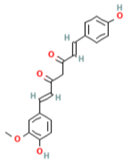	$C_{20}H_{18}O_{5}$	338.4	5469424	-
34	Curcumin	6951.2	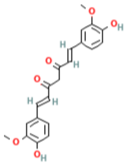	$C_{21}H_{20}O_{6}$	368.4	969516	-
35	Tetrahydroxyflavanone	241.0	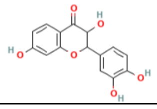	$C_{15}H_{12}O_{6}$	288.3	246330	-

Table S1. (continued).

No.	Compound name	Peak area	Structure	MF[1]	MW	PubChem CID[2]	Excluded reason[3]
36	Kaempferol 3,7-dimethoxy	2033.2	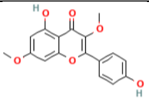	$C_{17}H_{14}O_6$	314.3	5318869	-
37	Wogonin	1690.7	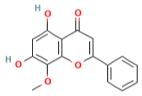	$C_{16}H_{12}O_5$	284.3	5281703	-
38	Rosmanol	1206.2	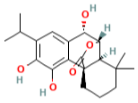	$C_{20}H_{26}O_5$	346.4	13966122	-
39	Epirosmanol	302.5	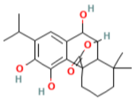	$C_{20}H_{26}O_5$	346.4	9884612	-
40	Octenylsuccinic anhydride	1684.2	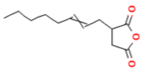	$C_{12}H_{18}O_4$	210.3	534543	NP (esterification agent)
41	20-HETE	4.5	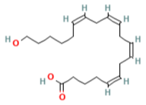	$C_{20}H_{32}O_3$	320.5	5283157	NP (fatty acids)
42	LysoPC(18:2)	237.8	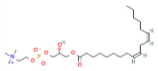	$C_{26}H_{50}NO_7P$	519.7	11005824	NP (fatty acids)
43	LysoPC(16:0)	275.8	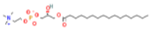	$C_{24}H_{50}NO_7P$	495.6	460602	NP (fatty acids)
44	Carnosic acid	2791.2	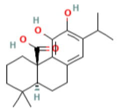	$C_{20}H_{28}O_4$	332.4	65126	-
45	LysoPC(18:1)	190.2	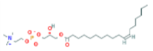	$C_{26}H_{52}NO_7P$	521.7	53480465	NP (fatty acids)
46	Pinusolide	460.5	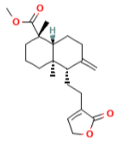	$C_{21}H_{30}O_4$	346.5	161721	-
	**Total**				46		
	**Excluded**				13		
	**Entry**				33		

The peak area value was the average of three replicates. The structure of compounds with molecular properties was retrieved from PubChem compounds database (https://pubchem.ncbi.nlm.nih.gov/). PBS, a plant-based multivitamin/mineral supplement; UPLC-Q-TOF-MS, ultra-high performance liquid chromatography-quadrupole time-of-flight mass spectrometry; MF, molecular formula; MW, molecular weight (g/mol); NS, not sufficient to specify a single compound; NP, not a phytochemical.

Table S2. List of oxidative stress-related biological processes.

Biological process
**Aging (GO:0007568)**
multicellular organism aging
cell aging
negative regulation of cell aging
positive regulation of cell aging
**Cell redox homeostasis (GO:0045454)**
cell redox homeostasis
**Response to redox state (GO:0051775)**
response to redox state
cellular response to redox state
**ER stress response (GO:0034976)**
response to endoplasmic reticulum stress
**Response to DNA damage checkpoint signaling (GO:0072423)**
response to G1 DNA damage checkpoint signaling
regulation of response to DNA damage checkpoint signaling
response to intra-S DNA damage checkpoint signaling
**DNA damage response (GO:0006974)**
signal transduction in response to DNA damage
signal transduction involved in DNA damage checkpoint
regulation of DNA damage checkpoint
negative regulation of DNA damage checkpoint
positive regulation of DNA damage checkpoint
mitotic DNA damage checkpoint
mitotic G1 DNA damage checkpoint
mitotic G2 DNA damage checkpoint
signal transduction involved in G2 DNA damage checkpoint
intra-S DNA damage checkpoint
negative regulation of mitotic DNA damage checkpoint
regulation of response to DNA damage stimulus
DNA damage response, signal transduction by p53 class mediator
regulation of DNA damage response, signal transduction by p53 class mediator
positive regulation of DNA damage response, signal transduction by p53 class mediator
negative regulation of DNA damage response, signal transduction by p53 class mediator
DNA damage response, signal transduction by p53 class mediator resulting in cell cycle arrest
DNA damage response, signal transduction by p53 class mediator resulting in transcription of p21 class mediator
positive regulation of DNA damage response, signal transduction by p53 class mediator resulting in transcription of p21 class mediator
DNA damage response, signal transduction resulting in transcription
positive regulation of response to DNA damage stimulus
intrinsic apoptotic signaling pathway in response to DNA damage
negative regulation of intrinsic apoptotic signaling pathway in response to DNA damage

Table S2. List of oxidative stress-related biological processes (continued).

Biological process
**DNA damage response (GO:0006974) (continued)**
positive regulation of intrinsic apoptotic signaling pathway in response to DNA damage
intrinsic apoptotic signaling pathway in response to DNA damage by p53 class mediator
regulation of intrinsic apoptotic signaling pathway in response to DNA damage by p53 class mediator
negative regulation of intrinsic apoptotic signaling pathway in response to DNA damage by p53 class mediator
positive regulation of intrinsic apoptotic signaling pathway in response to DNA damage by p53 class mediator
DNA damage induced protein phosphorylation
negative regulation of telomere maintenance in response to DNA damage
nucleotide-excision repair, DNA damage recognition
nucleotide-excision repair, DNA damage removal
regulation of transcription from RNA polymerase II promoter in response to UV-induced DNA damage
negative regulation of transcription from RNA polymerase II promoter in response to UV-induced DNA damage
**Response to oxidative stress (GO:0006979)**
regulation of response to oxidative stress
positive regulation of response to oxidative stress
negative regulation of response to oxidative stress
regulation of cellular response to oxidative stress
response to hydroperoxide
cellular response to hydroperoxide
response to lipid hydroperoxide
detection of oxidative stress
response to reactive oxygen species
regulation of response to reactive oxygen species
cellular response to reactive oxygen species
response to hydrogen peroxide
cellular response to hydrogen peroxide
regulation of hydrogen peroxide-induced cell death
positive regulation of hydrogen peroxide-induced cell death
negative regulation of hydrogen peroxide-induced cell death
negative regulation of hydrogen peroxide-induced neuron death
negative regulation of hydrogen peroxide-induced neuron intrinsic apoptotic signaling pathway
negative regulation of hydrogen peroxide-mediated programmed cell death
positive regulation of hydrogen peroxide-mediated programmed cell death
response to superoxide
cellular response to superoxide
cellular response to oxidative stress

Table S2. List of oxidative stress-related biological processes (continued).

Biological process
**Response to oxidative stress (GO:0006979) (continued)**
regulation of transcription from RNA polymerase II promoter in response to oxidative stress
positive regulation of transcription from RNA polymerase II promoter in response to oxidative stress
cell death in response to oxidative stress
negative regulation of oxidative stress-induced cell death
positive regulation of oxidative stress-induced cell death
intrinsic apoptotic signaling pathway in response to oxidative stress
regulation of oxidative stress-induced intrinsic apoptotic signaling pathway
negative regulation of oxidative stress-induced intrinsic apoptotic signaling pathway
positive regulation of oxidative stress-induced intrinsic apoptotic signaling pathway
intrinsic apoptotic signaling pathway in response to hydrogen peroxide
negative regulation of intrinsic apoptotic signaling pathway in response to hydrogen peroxide
neuron death in response to oxidative stress
regulation of oxidative stress-induced neuron intrinsic apoptotic signaling pathway
negative regulation of oxidative stress-induced neuron intrinsic apoptotic signaling pathway
positive regulation of oxidative stress-induced neuron intrinsic apoptotic signaling pathway
negative regulation of oxidative stress-induced neuron death
positive regulation of oxidative stress-induced neuron death
oxidative stress-induced premature senescence
cellular response to lipid hydroperoxide
**ROS metabolic process (GO:1901698)**
reactive oxygen species biosynthetic process
regulation of reactive oxygen species metabolic process
positive regulation of reactive oxygen species metabolic process
negative regulation of reactive oxygen species metabolic process
regulation of reactive oxygen species biosynthetic process
positive regulation of reactive oxygen species biosynthetic process
negative regulation of reactive oxygen species biosynthetic process
hydrogen peroxide metabolic process
regulation of hydrogen peroxide metabolic process
negative regulation of hydrogen peroxide metabolic process
hydrogen peroxide biosynthetic process
positive regulation of hydrogen peroxide biosynthetic process
negative regulation of hydrogen peroxide biosynthetic process
hydrogen peroxide catabolic process
positive regulation of hydrogen peroxide catabolic process
negative regulation of hydrogen peroxide catabolic process
superoxide metabolic process

Table S2. List of oxidative stress-related biological processes (continued).

Biological process
**ROS metabolic process (GO:1901698) (continued)**
regulation of superoxide metabolic process
removal of superoxide radicals
regulation of removal of superoxide radicals
superoxide anion generation
regulation of superoxide anion generation
positive regulation of superoxide anion generation
negative regulation of superoxide anion generation
**Response to nitrogen compound (GO:1901698)**
cellular response to nitric oxide
cellular response to reactive nitrogen species
**Protein oxidation (GO:0018158)**
protein oxidation
positive regulation of protein oxidation
**Lipid oxidation (GO:0034440)**
fatty acid oxidation
regulation of fatty acid oxidation
negative regulation of fatty acid oxidation
positive regulation of fatty acid oxidation
fatty acid omega-oxidation
regulation of lipoprotein lipid oxidation
negative regulation of lipoprotein lipid oxidation
NADH oxidation
**Transport (GO:0006810)**
lipid hydroperoxide transport
glutathione transport
glutathione transmembrane transport
**NADH oxidation (GO:0006116)**
NADH oxidation
**Sulfur compound metabolic process (GO:0006790)**
glutathione biosynthetic process
positive regulation of glutathione biosynthetic process
glutathione catabolic process
glutathione deglycation
glutathione derivative biosynthetic process
sulfur oxidation
sulfide oxidation, using sulfide:quinone oxidoreductase
**Regulation of oxidoreductase activity (GO:0051341)**
positive regulation of glutathione peroxidase activity
regulation of superoxide dismutase activity
positive regulation of superoxide dismutase activity

Table S2. List of oxidative stress-related biological processes (continued).

Biological process	
**Regulation of thioredoxin peroxidase activity (GO:1903123)**	
negative regulation of thioredoxin peroxidase activity by peptidyl-threonine phosphorylation	
**NADPH oxidation (GO:0070995)**	
NADPH oxidation	
**Heme oxidation (GO:0006788)**	
heme oxidation	
**Response to host defenses (GO:0052200)**	
response to defense-related host reactive oxygen species production	
**Total**	**141**

ER, endoplasmic reticulum; DNA, deoxyribonucleic acid; ROS, reactive oxygen species; NADH, nicotinamide adenine dinucleotide (NAD) + hydrogen; NADPH, nicotinamide adenine dinucleotide phosphate + hydrogen.

Table S3. List of inflammation-related biological processes.

Biological process
**Inflammatory response (GO:0006954)**
activation of plasma proteins involved in acute inflammatory response
acute inflammatory response
acute inflammatory response to antigenic stimulus
chronic inflammatory response
chronic inflammatory response to antigenic stimulus
cytokine production involved in inflammatory response
fever generation
histamine secretion by mast cell
inflammatory cell apoptotic process
inflammatory response to antigenic stimulus
leukocyte activation involved in inflammatory response
leukocyte chemotaxis involved in inflammatory response
leukocyte migration involved in inflammatory response
leukotriene production involved in inflammatory response
negative regulation of acute inflammatory response
negative regulation of acute inflammatory response to antigenic stimulus
negative regulation of acute inflammatory response to non-antigenic stimulus
negative regulation of chronic inflammatory response
negative regulation of chronic inflammatory response to antigenic stimulus
negative regulation of cytokine production involved in inflammatory response
negative regulation of inflammatory response
negative regulation of inflammatory response to antigenic stimulus
negative regulation of respiratory burst involved in inflammatory response
nitric oxide production involved in inflammatory response
positive regulation of acute inflammatory response
positive regulation of acute inflammatory response to antigenic stimulus
positive regulation of acute inflammatory response to non-antigenic stimulus
positive regulation of chronic inflammatory response
positive regulation of chronic inflammatory response to antigenic stimulus
positive regulation of chronic inflammatory response to non-antigenic stimulus
positive regulation of cytokine production involved in inflammatory response
positive regulation of fever generation
positive regulation of fever generation by positive regulation of prostaglandin secretion
positive regulation of histamine secretion by mast cell
positive regulation of inflammatory response
positive regulation of inflammatory response to antigenic stimulus
positive regulation of leukotriene production involved in inflammatory response
positive regulation of respiratory burst involved in inflammatory response
production of molecular mediator involved in inflammatory response

Table S3. List of inflammation-related biological processes (continued).

Biological process
**Inflammatory response (GO:0006954) (continued)**
regulation of acute inflammatory response
regulation of chronic inflammatory response
regulation of cytokine production involved in inflammatory response
regulation of fever generation
regulation of inflammatory response
regulation of vascular permeability involved in acute inflammatory response
**Cytokine production (GO:0001816)**
B cell cytokine production
cytokine production involved in immune response
dendritic cell cytokine production
interleukin-1 alpha production
interleukin-1 beta production
interleukin-1 production
interleukin-2 production
interleukin-3 production
interleukin-4 production
interleukin-5 production
interleukin-6 production
interleukin-8 production
interleukin-9 production
interleukin-10 production
interleukin-12 production
interleukin-13 production
interleukin-15 production
interleukin-17 production
interleukin-18 production
interleukin-21 production
interleukin-33 production
macrophage cytokine production
myeloid dendritic cell cytokine production
negative regulation of cytokine activity
negative regulation of cytokine production
negative regulation of dendritic cell cytokine production
negative regulation of interleukin-1 alpha production
negative regulation of interleukin-1 beta production
negative regulation of interleukin-1 production
negative regulation of interleukin-2 production
negative regulation of interleukin-3 production
negative regulation of interleukin-4 production

Table S3. List of inflammation-related biological processes (continued).

Biological process
**Cytokine production (GO:0001816) (continued)**
negative regulation of interleukin-5 production
negative regulation of interleukin-6 production
negative regulation of interleukin-8 production
negative regulation of interleukin-10 production
negative regulation of interleukin-12 production
negative regulation of interleukin-13 production
negative regulation of interleukin-17 production
negative regulation of interleukin-18 production
negative regulation of interleukin-23 production
negative regulation of macrophage cytokine production
negative regulation of macrophage inflammatory protein 1 alpha production
negative regulation of mast cell cytokine production
negative regulation of natural killer cell cytokine production
negative regulation of plasmacytoid dendritic cell cytokine production
negative regulation of T cell cytokine production
negative regulation of T-helper 2 cell cytokine production
negative regulation of tumor necrosis factor production
negative regulation of tumor necrosis factor superfamily cytokine production
positive regulation of cytokine activity
positive regulation of cytokine production
positive regulation of cytokine production involved in immune response
positive regulation of dendritic cell cytokine production
positive regulation of interleukin-1 alpha production
positive regulation of interleukin-1 beta production
positive regulation of interleukin-1 production
positive regulation of interleukin-2 production
positive regulation of interleukin-3 production
positive regulation of interleukin-4 production
positive regulation of interleukin-5 production
positive regulation of interleukin-6 production
positive regulation of interleukin-8 production
positive regulation of interleukin-10 production
positive regulation of interleukin-12 production
positive regulation of interleukin-13 production
positive regulation of interleukin-15 production
positive regulation of interleukin-16 production
positive regulation of interleukin-17 production
positive regulation of interleukin-18 production
positive regulation of interleukin-21 production

Table S3. List of inflammation-related biological processes (continued).

Biological process
**Cytokine production (GO:0001816) (continued)**
positive regulation of interleukin-23 production
positive regulation of interleukin-26 production
positive regulation of interleukin-33 production
positive regulation of macrophage cytokine production
positive regulation of macrophage inflammatory protein 1 alpha production
positive regulation of mast cell cytokine production
positive regulation of myeloid leukocyte cytokine production involved in immune response
positive regulation of natural killer cell cytokine production
positive regulation of T cell cytokine production
positive regulation of T-helper 1 cell cytokine production
positive regulation of T-helper 2 cell cytokine production
positive regulation of tumor necrosis factor (ligand) superfamily member 11 production
positive regulation of tumor necrosis factor production
regulation of B cell cytokine production
regulation of cytokine activity
regulation of cytokine production
regulation of cytokine production involved in immune response
regulation of dendritic cell cytokine production
regulation of interleukin-1 beta production
regulation of interleukin-1 production
regulation of interleukin-2 production
regulation of interleukin-4 production
regulation of interleukin-5 production
regulation of interleukin-6 production
regulation of interleukin-8 production
regulation of interleukin-10 production
regulation of interleukin-12 production
regulation of interleukin-15 production
regulation of interleukin-18 production
regulation of interleukin-19 production
regulation of interleukin-23 production
tumor necrosis factor production
regulation of tumor necrosis factor production
T cell cytokine production
T-helper 1 cell cytokine production
T-helper 2 cell cytokine production
regulation of T cell cytokine production

Table S3. List of inflammation-related biological processes (continued).

Biological process
**Response to cytokine (GO:0034097)**
cellular response to cytokine stimulus
cellular response to interleukin-1
cellular response to interleukin-2
cellular response to interleukin-3
cellular response to interleukin-4
cellular response to interleukin-6
cellular response to interleukin-7
cellular response to interleukin-8
cellular response to interleukin-11
cellular response to interleukin-13
cellular response to tumor necrosis factor
cytokine-mediated signaling pathway
interleukin-1-mediated signaling pathway
interleukin-2-mediated signaling pathway
interleukin-3-mediated signaling pathway
interleukin-4-mediated signaling pathway
interleukin-5-mediated signaling pathway
interleukin-6-mediated signaling pathway
interleukin-7-mediated signaling pathway
interleukin-8-mediated signaling pathway
interleukin-9-mediated signaling pathway
interleukin-11-mediated signaling pathway
interleukin-12-mediated signaling pathway
interleukin-13-mediated signaling pathway
interleukin-15-mediated signaling pathway
interleukin-17-mediated signaling pathway
interleukin-18-mediated signaling pathway
interleukin-21-mediated signaling pathway
interleukin-23-mediated signaling pathway
interleukin-27-mediated signaling pathway
interleukin-33-mediated signaling pathway
interleukin-35-mediated signaling pathway
negative regulation of cytokine-mediated signaling pathway
negative regulation of interleukin-1-mediated signaling pathway
negative regulation of interleukin-2-mediated signaling pathway
negative regulation of interleukin-4-mediated signaling pathway
negative regulation of interleukin-6-mediated signaling pathway
negative regulation of response to cytokine stimulus
negative regulation of tumor necrosis factor-mediated signaling pathway

Table S3. List of inflammation-related biological processes (continued).

Biological process	
**Response to cytokine (GO:0034097) (continued)**	
positive regulation of cytokine-mediated signaling pathway	
positive regulation of interleukin-1-mediated signaling pathway	
positive regulation of interleukin-4-mediated signaling pathway	
positive regulation of interleukin-6-mediated signaling pathway	
positive regulation of interleukin-17-mediated signaling pathway	
positive regulation of interleukin-18-mediated signaling pathway	
positive regulation of response to cytokine stimulus	
positive regulation of tumor necrosis factor-mediated signaling pathway	
regulation of cytokine-mediated signaling pathway	
regulation of interleukin-1-mediated signaling pathway	
regulation of tumor necrosis factor-mediated signaling pathway	
response to interleukin-1	
response to interleukin-2	
response to interleukin-4	
response to interleukin-6	
response to interleukin-9	
response to interleukin-11	
response to interleukin-12	
response to interleukin-13	
response to interleukin-15	
response to interleukin-18	
response to tumor necrosis factor	
tumor necrosis factor-mediated signaling pathway	
**Response to prostaglandin (GO:0034694)**	
cellular response to prostaglandin D stimulus	
cellular response to prostaglandin E stimulus	
cellular response to prostaglandin stimulus	
positive regulation of prostaglandin-E synthase activity	
positive regulation of prostaglandin-endoperoxide synthase activity	
response to prostaglandin E	
**Prostaglandin metabolic process (GO:0006693)**	
cyclooxygenase pathway	
negative regulation of prostaglandin biosynthetic process	
positive regulation of prostaglandin biosynthetic process	
prostaglandin biosynthetic process	
regulation of prostaglandin biosynthetic process	
**Total**	**226**
